# Total MRI load of cerebral small vessel disease and cognitive ability in older people

**DOI:** 10.1016/j.neurobiolaging.2015.06.024

**Published:** 2015-10

**Authors:** Julie Staals, Tom Booth, Zoe Morris, Mark E. Bastin, Alan J. Gow, Janie Corley, Paul Redmond, John M. Starr, Ian J. Deary, Joanna M. Wardlaw

**Affiliations:** a Department of Neurology and Cardiovascular Research Institute Maastricht, Maastricht University Medical Centre, Maastricht, The Netherlands; b Brain Research Imaging Centre, Department of Clinical Neurosciences, University of Edinburgh, Edinburgh, UK; c Department of Psychology, University of Edinburgh, Edinburgh, UK; d Centre for Cognitive Ageing and Cognitive Epidemiology, University of Edinburgh, Edinburgh, UK; e Department of Psychology, School of Life Sciences, Heriot-Watt University, Edinburgh, UK

**Keywords:** Cognitive aging, MRI, Cerebrovascular disease, Cerebral small vessel disease, White matter hyperintensities, Cerebral microbleeds, Lacunes, Perivascular spaces

## Abstract

Cerebral small vessel disease (SVD) may cause cognitive dysfunction. We tested the association between the combined presence of magnetic resonance imaging (MRI) features of SVD and cognitive ability in older age. Cognitive testing and brain MRI were performed in 680 older participants. MRI presence of lacunes, white matter hyperintensities, microbleeds, and perivascular spaces were summed in a score of 0–4 representing all SVD features combined. We also applied latent variable modeling to test whether the 4 MRI features form a unitary SVD construct. The SVD score showed significant associations with general cognitive ability. Latent variable modeling indicated that the 4 MRI markers formed a unitary construct, which showed consistent associations with cognitive ability compared with the SVD score. Total MRI load of SVD is associated with lower general cognitive ability in older age. The total SVD score performed consistently with the more complex latent variable model, suggesting validity and potential utility in future research for determining total SVD load.

## 1 Introduction

Cerebral small vessel disease (SVD) is a prevalent disease in older people. The underlying pathogenesis is still debated, and classic vascular risk factors are only one contributing variable (Wardlaw et al., 2013). SVD affects the deep perforating vessels in the brain, which causes brain damage that can be seen on magnetic resonance imaging (MRI). White matter hyperintensities (WMH) and lacunes are the most acknowledged MRI features (Debette and Markus, 2010, Vermeer et al., 2007), whereas microbleeds and perivascular spaces have more recently been recognized as consequences of SVD (Wardlaw et al., 2013).

Cerebral SVD is a major cause of cognitive dysfunction in older age (Gorelick et al., 2011). Many studies have demonstrated an association between WMH and poorer cognitive functioning (Debette and Markus, 2010, de Groot et al., 2000, Pantoni et al., 2007, Van der Flier et al., 2005). Other MRI markers of SVD have also all individually shown negative associations with cognition (Maclullich et al., 2004, Patel et al., 2013, Poels et al., 2012, Vermeer et al., 2003). These MRI markers often occur together, but the idea of addressing all features combined as a unitary measure of SVD has only gained attention recently (Brenner et al., 2008, Huijts et al., 2013, Klarenbeek et al., 2013). A total SVD measure might better enclose the global effect of SVD on the brain than the individual MRI features separately.

Huijts et al. (2013) devised a score of overall SVD load by summing all 4 MRI features and found an association with poorer cognitive function (Huijts et al., 2013) in a small study in a mixed sample of patients with lacunar stroke and hypertension. More validity to this total SVD score was recently added in a publication that showed associations with previously described well-known risk factors for individual SVD features, such as age, hypertension, and lacunar stroke (Staals et al., 2014). However, the results on total SVD load and cognitive function have not been validated, nor has the utility of this total SVD score been tested in generally healthy populations where SVD features might be less frequent. Furthermore, whether these 4 MRI SVD features jointly really indicate 1 underlying latent disease construct is untested. A latent variable is defined as a variable that cannot be directly measured (e.g., SVD) but is inferred through the measurements of a number of observable variables (e.g., MRI features).

In a large community-dwelling sample of older people, we used latent variable modeling to test whether the 4 MRI features form a unitary SVD construct. This would contribute to validating a simple and practical summed score for total SVD load. We then tested the association between cognitive ability and total SVD load, both as a latent and as a summed variable, and determined consistency across the 2 different SVD variable approaches. Finally, we explored whether total SVD score remained predictive independent of WMH, as WMH have been most regularly associated with cognitive functioning in published research (Valdés Hernández Mdel et al., 2013) and could solely drive the results.

## 2 Methods

### 2.1 Participants

Participants in this study came from the Lothian Birth Cohort 1936 (LBC1936), which has been described in detail before (Deary et al., 2007). In short, participants were all born in 1936, mostly reside in the Edinburgh area (Lothian) of Scotland, UK, and most had completed an intelligence test (Moray House Test number 12) at age 11 years. They were recruited into a longitudinal study of aging at a mean age of 70 years (Wave 1: *n* = 1091). Between 2007 and 2010, at the age of about 73 years (Wave 2), 700 participants underwent brain MRI scanning and cognitive testing. For the present study, complete MRI data were available in 680 participants. Demographics and self-reported medical history were taken in a standardized interview at Wave 2 (Deary et al., 2007). The study was approved by the Scotland and Lothian Research Ethics Committee, and all participants gave written informed consent.

### 2.2 Cognitive testing

Age 11 IQ was computed from the scores on the validated Moray House Test (Deary et al., 2004a, Deary et al., 2007) that was taken in 1947. Age 11 IQ correlates well with estimates of prior cognitive ability that were taken at Wave 2 (National Adult Reading Test, *r* = 0.7; Wechsler Test of Adult Reading, *r* = 0.7) (Dykiert and Deary, 2013).

Participants completed an extensive battery of cognitive tests at Wave 2. A full description of the background of these tests and assessment methods can be found elsewhere (Deary and Der, 2005, Deary et al., 2007). Here we use raw scores from 5 subtests of the Wechsler Adult Intelligence Scale-III (Block Design, Matrix Reasoning, Digit Symbol Coding, Symbol Search, Letter-Number Sequencing), 6 subtests of the Wechsler Memory Scale-III (Logical Memory immediate and delayed recall, Verbal Paired Associates, Digit Span Backwards, Spatial Span forward and backward recall), tests of vocabulary that are often used to estimate prior ability (National Adult Reading Test and Wechsler Test of Adult Reading), simple and choice reaction time tasks (Deary et al., 2001, Deary et al., 2010), and a test of the efficiency of visual processing called inspection time (Deary et al., 2004b, Deary et al., 2010).

From the individual test scores, we generated scores for general cognitive ability (*g*) and 2 specific ability domains that are also sensitive to aging—processing speed and memory—using confirmatory bi-factor modeling. A full description and rationale for these models in the LBC1936 have been described previously (Booth et al., 2013). Briefly, performance on any single cognitive ability subtest is influenced by both an individual's level of *g* and their level of specific ability domains. Thus, if one computes a score for a specific ability without first removing the proportion of variance associated with *g*, associations with external variables will be confounded by the 2 sources of ability variance. Bi-factor modeling partitions the variance of single subtests into variance that is general, and variance which is specific, thus yielding scores for *g* and for specific ability domains which are independent of *g*.

### 2.3 Brain MRI acquisition

Full details of the brain imaging protocol have been described previously (Wardlaw et al., 2011). Participants were all scanned on a General Electric 1.5 T clinical MRI scanner. For this study, we used axial T2, T2*, fluid-attenuated inversion recovery (FLAIR), and T1-weighted sequences.

### 2.4 MRI rating and composition of SVD scale

Images were rated by a certified and registered neuroradiologist (Z.M.) for the presence of lacunes, WMH, microbleeds, and perivascular spaces, but blind to all other information. The rating protocol, using validated visual scales, has been published (Wardlaw et al., 2011). Uncertain readings and a 20% random sample were checked independently by a second experienced, certified, and registered neuroradiologist (J.M.W.) and consensus was made on disagreements. The intraclass correlation coefficient for the rating of WMH was 0.96 (Aribisala et al., 2014); intra- and inter-rater kappa statistics for perivascular spaces ranged from 0.8 to 0.9 (Aribisala et al., 2014). Separately, an ordinal SVD score of 0 to 4 was created, representing the total MRI load of SVD, by counting the presence of each of these 4 MRI features, as described below (Huijts et al., 2013, Klarenbeek et al., 2013, Staals et al., 2014). The SVD score was developed after the image rating which precludes any influence on image rating.

Periventricular and deep WMH were rated on the Fazekas scale (each 0–3) using FLAIR- and T2-weighted sequences (Fazekas et al., 1987). One point was awarded on the SVD scale when (early) confluent deep WMH (Fazekas score 2 or 3) and/or irregular periventricular WMH extending into the deep white matter (Fazekas score 3) were present. This cut-point was justified and tested in earlier studies (Klarenbeek et al., 2013, Staals et al., 2014). Lacunes were defined as small (<15 mm), subcortical lesions of similar signal to CSF, i.e., increased signal on T2-weighted, decreased signal on FLAIR and T1-weighted images. One point on the SVD scale was awarded when 1 or more lacunes were present. Microbleeds were defined as small (<5 mm), homogeneous, round foci of low signal intensity on T2*-weighted images in cerebellum, brain stem, basal ganglia, white matter, or cortico-subcortical junction that were not simply vessel flow voids or other artifacts. The presence of 1 or more microbleeds gave 1 point on the SVD scale. Basal ganglia perivascular spaces were defined as small (<3 mm) punctate hyperintensities on T2-weighted images, and they were rated on a previously described semi-quantitative scale from 0 to 4 (Doubal et al., 2010). One point on the SVD scale was awarded when moderate to severe (grade 2–4) basal ganglia perivascular spaces were present.

Finally, we also rated cerebral atrophy at deep (enlargement of the ventricles) and superficial (enlargement of the sulci) levels, both on a 6-point scale based on age-specific reference templates, summed to a 2–12 atrophy score (Farrell et al., 2009).

### 2.5 Latent variable modeling

We used structural equation modeling (SEM) to assess the plausibility of a latent SVD variable. Latent variables are estimated from the common variance between a set of inter-correlated indicators (here SVD markers), partitioning common variance from error or unique variance. Furthermore, the SEM approach models the full range of each of the constituent variables, not merely their presence or absence (as is the case when continuous variables are dichotomized). Thus, the use of the latent modeling approach can contribute to the validation of the simple sum score if it confirms that the individual MRI markers are underpinned by a unitary latent SVD construct, and that associations with cognitive abilities are consistent across the 2 different modeling approaches.

To account for the non-normal distributions of the data, deep and periventricular WMH and perivascular spaces were modeled as ordered categorical variables, and lacunes and microbleeds were modeled as count variables. A single latent SVD variable was estimated with its variance fixed to 1 to allow identification of the model. Models were estimated using maximum likelihood with χ^2^ correction and numerical integration.

### 2.6 Statistical analysis

Clinical and radiological characteristics are presented as numbers with percentages, mean with standard deviation (SD) or median (range). The association between SVD (independent variable) and cognitive ability (*g*, processing speed or memory; dependent variable) was analyzed using linear regression analysis. We performed the analyses with the ordinal 0-4 SVD scale in IBM SPSS 20 Statistics, and with the continuous latent SVD variable in MPlus 6.0.

In the first model, we included only SVD scale score. In the second model, we included age, sex, age 11 IQ, vascular health variables (history of transient ischemic attack (TIA) and/or stroke, cardiovascular disease, diabetes, hypertension, ever smoked, body mass index, total cholesterol, alcohol use) and rating of cerebral atrophy. Prior ability (age 11 IQ) was included as it strongly influences cognitive ability in later life. There were 35 missing observations for age 11 IQ and 24 missing observations for cholesterol, giving *n* = 622 in the second model.

We repeated these analyses using the latent SVD variable modeled using SEM. The purpose of this analysis was to assess the degree of attenuation of association caused by dichotomizing continuous variables in the production of the ordinal SVD score. Here, our primary interest concerns differences in the parameter estimates (Beta) across models, not the nominal *p* values of the effects.

To test whether results were driven exclusively by WMH, we repeated the analyses excluding WMH from the ordinal SVD scale (giving a 0–3 score).

Lastly, although the primary focus of the present study was the validation of the SVD score, we also present results of substantive interest. As such, we carried out 2 additional sensitivity checks. We repeated our main analyses after exclusion of participants with a prior TIA or stroke, and participants with a Mini-Mental State Examination (MMSE)<26, indicating possible dementia.

All results are shown as unstandardized betas. Our 3 models include 12 predictors, resulting in a Bonferroni adjusted *p* value for statistical significance of 0.0014. Where appropriate, results are discussed with respect to both the unadjusted and adjusted *p* values; however, we note that our primary interest is in the magnitude of the parameter estimates across models.

## 3 Results

### 3.1 Participants

Table 1 presents clinical and radiological characteristics of all 680 participants. According to the definitions used for the SVD scale, perivascular spaces were most prevalent, followed by WMH, microbleeds, and lacunes. Scores on the SVD scale are presented in Table 2. Most participants achieved 0 or 1 point on the SVD score, whereas scoring all 4 MRI features was very infrequent.

### 3.2 Cognitive function and SVD score

Table 3 (top panel) presents the results of linear regression models of associations between SVD score and cognitive abilities. Having more MRI markers of SVD, represented by a higher SVD score, was associated with lower *g*: unstandardized beta −0.131, *p* = 0.0013. This means that for each point increase in SVD score, *g* is 0.141 SD lower (SD of *g* in this subset of 680 participants was 0.93). This association attenuated after controlling for age, sex, prior cognitive ability (age 11 IQ), vascular health status, and cerebral atrophy: beta −0.082, *p* = 0.017. SVD score was not associated with processing speed or memory in the unadjusted model; after full adjustment, the association between SVD score and memory appeared to be significant with *p* = 0.032, which, however, was not significant after Bonferroni correction.

### 3.3 Cognitive function and latent SVD variable

**Fig. 1:**
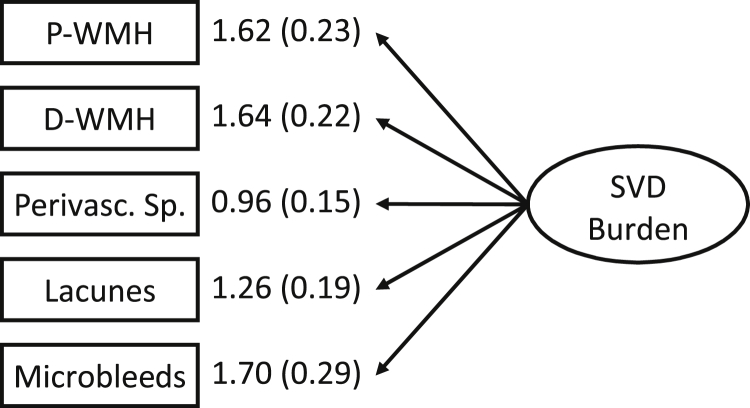
Structural equation modeling diagram for the SVD latent variable construct. All factor loadings are unstandardized and significant (*p <* 0.001). Unstandardized loadings are interpreted as the raw unit increase in the indicator per standard deviation (SD) increase in the latent construct, e.g. an 1 SD increase in SVD latent variable leads to an increase of approximately 1.7 visible microbleeds. P-WMH, periventricular WMH Fazekas rating; D-WMH, deep WMH Fazekas rating; Perivasc.Sp. perivascular spaces.

Fig. 1 shows the SEM diagram for the SVD latent variable construct. All individual marker variables loaded significantly on the latent SVD construct which confirms that the individual features contribute to the SVD latent variable. All loadings are unstandardized as it is not possible to standardize count variables (number of lacunes and microbleeds). The factor loadings reported in Fig. 1 can be interpreted as the raw unit increase in the indicators per SD increase in the latent variable.

The results for the latent SVD analyses are shown in Table 3 (middle panel). There was a significant association for *g* in the unadjusted models. Importantly, as can be seen from a comparison of the middle to top panel, the pattern of results is highly similar across the SVD scale and latent SVD analyses. The magnitudes of effects are larger in the latent SVD model, as would be expected. In the case of *g*, the parameter estimates are highly consistent, and the size of attenuation through the addition of covariates is also equivalent.

### 3.4 Sensitivity analysis

The above analyses were re-run after excluding participants with a self-reported history of TIA or stroke (*n* = 47). For the speed and *g* models, this resulted in no change in the key associations between SVD and cognitive ability. In the latent models for memory, a 0.016 increase in parameter estimate occurred. Secondly, analysis excluding cases with MMSE scores <26 (*n* = 25) resulted in changes in parameter estimates of up to 0.032, across the latent and SVD score models. In the SVD scale, this resulted in a nonsignificant association with *g* in the fully adjusted model.

### 3.5 Cognitive function and SVD excluding the impact of WMH

As shown in Table 3 (bottom panel), removing WMH from the construction of the SVD score resulted in no substantive changes in interpretation for *g*, although the *p*-values could not withstand Bonferroni correction for multiple testing. SVD score without WMH was not associated with processing speed or memory.

## 4 Discussion

Greater total SVD load on MRI was associated with lower general cognitive ability (*g*) in a population of healthy older participants. This association remained similar when WMH were excluded. Latent variable modeling provided support for the combination of different MRI features into 1 overall SVD score. The latent variable SVD measure showed consistent associations with *g* when compared with a simple-sum SVD score, but there was a small degree of variability in the magnitudes of the parameter estimates for speed and memory.

Cerebral SVD is considered to have a significant influence on cognitive ability and is among the most common causes of cognitive impairment and dementia (Gorelick et al., 2011). These conclusions are based on numerous studies that have examined single MRI features of SVD only. Analyzing the combined presence of these MRI features as 1 disorder was recently tried in a small study in a mixed population of patients with a high prevalence of SVD (patients with lacunar stroke and hypertension) (Huijts et al., 2013). We confirm and extend these results in a large population of healthy older participants.

The results of the latent variable modeling analyses confirm that the specified MRI features are jointly indicative of an underlying overall SVD state. Dichotomization using cut-point criteria, as utilized in creating the simple SVD scale, is necessary and sensible for a practical clinical scale, but in a research context it leads to a loss of information and power. Some evidence of this effect was found in comparison of the latent SVD analysis to the simple sum score. In most cases, our results showed larger effects in the latent models, however these differences were small. It is important to note that we are not suggesting that the simple SVD scale is ideal as a measure of SVD; factors such as the number and location of the individual MRI features are also known to contribute to cognitive dysfunction (Benisty et al., 2009, Carey et al., 2008, Poels et al., 2012) and each individual feature may have different weights of effects. Furthermore, there are other SVD-associated features, such as microinfarcts (not visible on conventional MRI) and cerebral atrophy, that also contribute to cognitive aging (Launer et al., 2011). These should be tested in future studies. Nevertheless, our results imply a cumulative effect of different SVD features on general cognitive ability, possibly reflecting more extensive vascular pathology in the brain (Deramecourt et al., 2012, Smallwood et al., 2012), that can be estimated using a simple-sum SVD score.

Effects of SVD on cognitive aging are generally found more in information processing speed and executive function than in memory (Debette and Markus, 2010, Pantoni, 2010, Pantoni et al., 2007, Patel et al., 2013, Poels et al., 2012). We did not find an association between total SVD load and processing speed. The absence of a clear association between total SVD load and processing speed in our study may relate to a more heavy reliance of this cognitive ability on 1 single MRI feature, especially WMH, maybe because different features may disrupt brain tissue and cognitive pathways in different locations or extents. Indeed, we found no association between SVD score *excluding* WMH and processing speed, whereas an association was present between WMH (Fazekas score) separately and processing speed (results not shown). More extensive analyses using quantitative WMH measurements and cognitive ability in this cohort have been published recently (Valdés Hernández Mdel et al., 2013). Assessing all SVD features together may better capture the global effect on the brain. Furthermore, most other studies did not produce scores on specific cognitive domains that were independent of *g*; therefore the associations between SVD load and processing speed in other studies may have been determined at least in part by the effect of *g* (Booth et al., 2013).

We did not find an association between SVD load and memory. The 1 significant *p* value in the fully adjusted model of SVD scale and memory was not a consistent finding: it was nonsignificant after Bonferroni-correction, nonsignificant after exclusion of WMH and was not found in the latent variable model. In the cognitive profile of SVD, memory is generally believed to be relatively spared; however, a recent review suggested that impaired cognition in patients with symptomatic small vessel stroke is less selective than previously thought and involves all major cognitive domains (Edwards et al., 2013). As noted, we find little evidence for this in the present study where we used memory scores independent of *g*.

Strengths of our study are the population-based setting, large sample size, detailed cognitive testing, and correction for vascular health status. Furthermore, controlling for age 11 IQ is especially important as cognitive ability from youth correlates with education level and occupational attainment and is a strong determinant of cognitive ability in older age (Plassman et al., 1995). By controlling current cognitive ability for prior cognitive ability, our results can be interpreted as an association of SVD with lifetime change in cognitive ability. Finally, the age-homogeneity of a birth cohort (partly) erases the important confounder effect of age on cognitive ability, although the age effect often is that strong that adjustment is still required. However, generalizability of our results to other age groups might be limited.

An interesting finding in the present study was the loss of significant effect of SVD on *g* in models removing participants with MMSE <26. There are a number of possible explanations of this finding. Firstly, it may be that the significant association seen in the whole sample were being driven by this group of participants. To the extent that low MMSE scores may be indicative of mild cognitive impairment or incipient dementia, this may be a clinically interesting observation. However, it is also possible that the loss of significance was because of the decrease in statistical power from the drop in sample size. Given the generally small effects in the present study, this may also be plausible.

Study limitations include the cross-sectional design. Longitudinal studies exploring progression of SVD and within-old-age decline of cognitive ability in specific domains would be of value. We did not cover all cognitive domains. However, information processing speed is considered one of the most affected cognitive abilities in SVD, may mediate age-related changes in other cognitive abilities including some aspects of memory (Finkel et al., 2007, Zimprich and Martin, 2002), and is therefore valuable to study. SVD comprises several vascular pathologies, such as intrinsic arteriolar disease and amyloid angiopathy (Pantoni, 2010). However, they produce similar features of brain damage on imaging, and our approach was to compile the overall brain damage resulting from SVD, not the underlying pathways. Further testing of the total SVD score at other ages in more diseased cohorts and in participants with specific diseases is warranted.

In conclusion, we applied 2 measures of total MRI load of SVD: a simple, pragmatic sum score and another derived from latent variable modeling. We found an association with lower general cognitive ability in a large general-population sample of older people. Future studies should examine whether overall SVD-related brain damage predicts cognitive decline within old age more sensitively than individual SVD features, and whether therapeutic interventions can ameliorate this.

## Disclosure statement

Julie Staals was supported by the Academic Fund of Maastricht University Medical Centre. Joanna M. Wardlaw was supported by the Scottish Funding Council through the SINAPSE Collaboration (http://www.sinapse.ac.uk). The rest of the authors report no conflicts of interest.

## Figures and Tables

**Table 1:** Participant characteristics

Clinical and radiological variables	All participants, N= 680
Age, mean (SD), years at MRI scanning	72.7 (0.7)
Male sex, No. (%)	359 (52.8)
History of TIA or stroke, No. (%)	47 (6.9)
Cardiovascular disease, No. (%)	183 (26.9)
Diabetes, No. (%)	70 (10.3)
High blood pressure, No. (%)	334 (49.1)
SBP, mean (SD), mmHg^a^	148.9 (19.0)
DBP, mean (SD), mmHg^a^	78.1 (9.6)
BMI, mean (SD)	27.9 (4.5)
Total cholesterol, mean (SD), mmol/L^b^	5.1 (1.1)
Smoking, ever, No. (%)	364 (53.5)
Alcohol use, mean (SD), units/week	10.5 (14.3)
MRI markers^c^	
WMH, No. (%)	154 (22.6)
Perivascular spaces, No. (%)	276 (40.6)
Microbleeds, No. (%)	79 (11.6)
Lacunes, No. (%)	33 (4.9)
WMH Fazekas score, median (range)	
Periventricular	1 (0–3)
Deep	1 (0–3)
Cerebral atrophy score, median (range)	7 (2–12)

Key: BMI, body mass index; DBP, diastolic blood pressure; MRI, magnetic resonance imaging; SBP, systolic blood pressure; SD, standard deviation; TIA, transient ischemic attack; WMH, white matter hyperintensities.

a Mean of 3 sitting blood pressure measurements.

b 24 (3.5%) missing.

c Defined as in the simple SVD scale.

**Table 2:** Cerebral small vessel disease score

SVD score, no. (%)	All participants, N = 680
0	302 (44.4)
1	249 (36.6)
2	98 (14.4)
3	27 (4.0)
4	4 (0.6)

Key: SVD, small vessel disease.

**Table 3:** Linear regression models of associations between cognitive abilities and SVD

	N	*g* unstandardized beta (SE)	*p*	Processing speed unstandardized beta (SE)	*p*	Memory unstandardized beta (SE)	*p*
SVD scale	680	−0.131 (0.041)	0.001	−0.062 (0.036)	0.084	−0.063 (0.037)	0.089
+ age, sex, IQ11, vasc. health, cerebral atrophy	622	−0.082 (0.034)	0.017	−0.039 (0.038)	0.306	−0.084 (0.039)	0.032
Latent SVD	680	−0.165 (0.048)	0.001	−0.117 (0.041)	0.004	−0.034 (0.041)	0.412
+ age, sex, IQ11, vasc. health, cerebral atrophy	622	−0.085 (0.045)	0.061	−0.083 (0.041)	0.054	−0.049 (0.042)	0.243
SVD scale without WMH	680	−0.143 (0.054)	0.008	−0.045 (0.047)	0.339	−0.077 (0.049)	0.116
+ age, sex, IQ11, vasc. health, cerebral atrophy	622	−0.101 (0.045)	0.025	−0.022 (0.049)	0.654	−0.070 (0.051)	0.170

Bonferroni adjusted *p*-value for statistical significance of 0.0014.

Key: IQ11, IQ at age 11 (prior cognitive ability); SE, standard error; SVD, small vessel disease; total, cholesterol; vasc. health, vascular health status (history of TIA/stroke, cardiovascular disease, diabetes, hypertension, ever smoking, body mass index, total cholesterol, alcohol use).
